# Resident Opinions on Image Guidance for External Ventricular Drain Placement: A National Survey

**DOI:** 10.1227/neuprac.0000000000000097

**Published:** 2024-08-05

**Authors:** Thomas Noh, Parikshit Juvekar, Gina Watanabe, Alexandra J. Golby

**Affiliations:** * John A Burns School of Medicine, University of Hawaii, Honolulu, Hawaii, USA;; ‡ Department of Neurological Surgery, Brigham and Women's Hospital, Harvard Medical School, Boston, Massachusetts, USA

**Keywords:** Image guidance, Resident opinions, Survey, External ventricular drain, Neuronavigation

## Abstract

**BACKGROUND AND OBJECTIVES:**

Low-quality data on image-guided external ventricular drain (EVD) accuracy are in large part due to a lack of widespread usage of this system for EVD placement. The potential disconnect between user preferences and existing technologies should be explored to guide future developments. The goal of this study was to survey neurosurgical residents regarding their EVD practices and determine the acceptable amount of setup time for an ideal neuronavigation system.

**METHODS:**

A 4-question survey was sent to approximately 1512 residents at 108 Acreditation Council for Graduate Medical Education–approved medical doctor neurosurgical training programs in the United States. The responses were received electronically, tabulated, and analyzed using descriptive statistics.

**RESULTS:**

A total of 130 respondents (9%) completed the survey, reflecting the highest number of neurosurgical resident respondents in an electronic qualitative survey of EVD practices thus far. Residents were willing to accept 6.39 min (SD = 3.73 min) on average for the setup of a bedside EVD image guidance system. The majority chose to use image guidance during EVD placement for cases of narrow slit-like ventricles (86.92%) over intraventricular hemorrhage (13.08%) and hydrocephalus (0%). A total of 90% of all resident respondents misplaced at least 1 EVD with 74% of post-graduate year–7 respondents misplacing more than 3 EVDs in their career. A total of 88.46% of respondents deemed more than a single pass as acceptable.

**CONCLUSION:**

Future EVD neuronavigation technologies should focus on achieving rapid registration times. These systems may be prioritized for patients with anatomic distortions. Current resident attitudes are accepting multiple EVD passes, likely because of the inherent limitations of the traditional freehand approach. Efforts should be made to encourage the best course for the patient.

**ABBREVIATIONS:**
AR: augmented reality
EVD: external ventricular drain.; PGY, post-graduate year.

External ventricular drain (EVD) placements are among the most common neurosurgical procedures performed, with an annual estimate of approximately 25 000 ventriculostomies conducted in the United States.^1^ Although deemed a relatively simple procedure,^2^ the standard method of a freehand pass has garnered criticism in the wake of reported misplacements,^3,4^ especially in patients with trauma.^5^ Each cannulation attempt delays a life-saving procedure in the acute setting and increases risk of infection and hemorrhage.^6-8^ Inadvertent positioning of the catheter tip into brain parenchyma or an unsatisfactory location within the ventricles or repeated contact with blood and clotting factors may obstruct the EVD and prompt EVD replacement. The cost of EVD complications is substantial with EVD replacement ranging from $1300 to $3200 and EVD-associated infection incurring $22 360 to $55 900 per patient.^2,9^ Surveys that reveal self-reported catheter misplacement and an unsettling range of 2 to 20 witnessed freehand passes have expedited the search for image guidance, particularly for patients with anatomic ventricular distortions.^10,11^

In response, a number of technological adjuncts have been developed to improve EVD placement accuracy and minimize complications. Mobile applications,^12-14^ electromagnetic navigation,^15-18^ mixed reality,^19^ and ultrasound^20-22^ are a few among many technologies that are evolving to guide EVD placement. However, the true efficacy of image guidance for EVD insertion is unclear. In 2016, the Neurocritical Care Society released a consensus statement on EVD insertion and suggested considering image guidance for cases of small or distorted ventricles.^23^ However, they note that there are low-quality data on image-guided EVD accuracy and a lack of meaningful comparison groups. Of 21 institutional EVD practices analyzed in a binational multicenter study, image guidance usage rates for EVD insertions ranged from 0% in 7 units to 100% in 1 unit for an average of 19.6%.^24^

O'Neill et al^2^ surveyed neurosurgeons across the country and found that 34% of practicing neurosurgeons and 27% of residents would never use an image guidance system. When the question was extended to include time, 93% of practicing and training surgeons said that they would not use image guidance if it added more than 10 minutes to the procedure. There were, however, no qualitative questions asked such as the size of the ventricles or the presence of shift. In addition, there was and still currently is no available workflow that can achieve image guidance capability in under 10 minutes.

Neurosurgery residents offer a unique opportunity to study surgeons. They have measurable levels of expertise and a homogeneous task of providing neurosurgical care on the front lines. They are also formulating opinions through experiences and could assumedly represent the least biased opinions with more novice humility. Thus, we performed a cross-sectional study of neurosurgical resident opinions regarding the use of image guidance for EVD placement.

## METHODS

**FIGURE 1.**
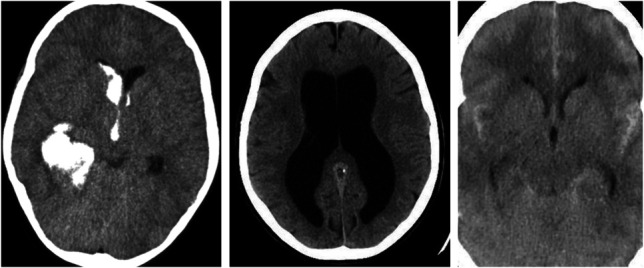
Case 1 (left), Case 2 (middle), and Case 3 (right) depicting intraventricular hemorrhage, hydrocephalus, and narrow slit-like ventricles, respectively. Respondents were asked to select which case that they would find image guidance helpful for and were presented with these single axial computed tomography slices alone.

A questionnaire was created using the XM platform survey tool (Qualtrics) that queried: the respondent's training level (post-graduate year [PGY]-1 to PGY-7), the number of EVDs misplaced by the respondents through their career, and the acceptable number of passes for EVD placement per the respondent. They were also asked a hypothetical question regarding the acceptable amount of time they would add to their workflow if they were provided with an autoregistering-image guidance system and asked which of the 3 provided cases (single axial computed tomography [CT] slice) (Figure 1) the respondent would opt for image guidance. The term “misplacement” was not defined in the survey. Cases 1, 2, and 3 depicted intraventricular hemorrhage in the right lateral ventricle, hydrocephalus, and narrow slit-like ventricles, respectively. Images were obtained from public domain images using a Google search, and thus, Institutional Review Board approval was not required.

This survey (see **Supplemental Digital Content 1**, http://links.lww.com/NS9/A30) was developed using the Checklist for Reporting Results of Internet E-Surveys (CHERRIES) checklist which is specific to web-based surveys^25^ and was Institutional Review Board–approved (#2018P001326). No consent was required as the study presented minimal risk. The survey was developed with little time commitment (1-minute) and distributed by chief residents to encourage participation by junior residents. Cookies and IP addresses were used to identify unique visitors but were not kept or analyzed for any demographic information. If a questionnaire was not complete, the data were not included.

**FIGURE 2.**
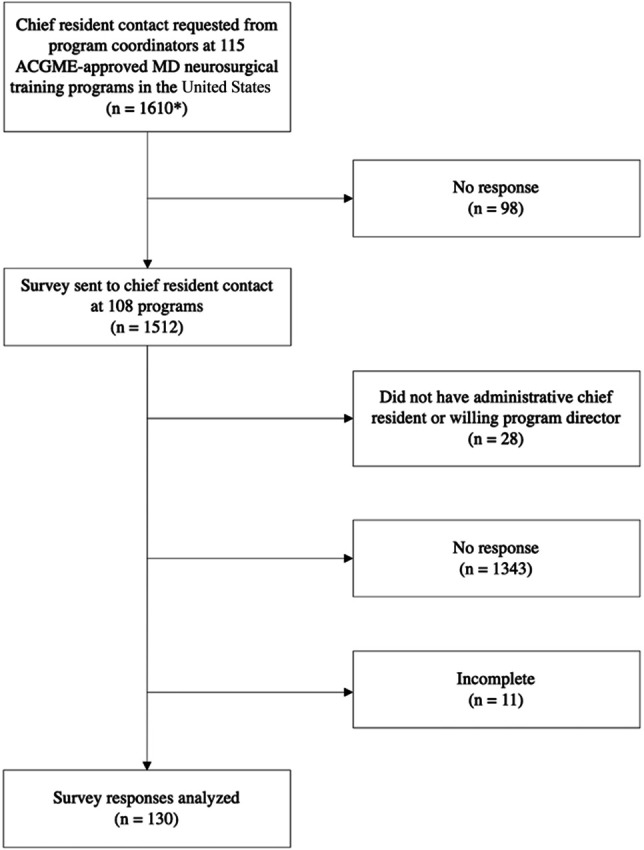
Flowchart of survey distribution. *The approximate number of residents is given in parentheses and is based on the National Resident Matching Program 2020 estimate of 14 residents per program.

The survey was administered through the Qualtrics program and was distributed via e-mail. First, program coordinators were contacted for administrative chief resident e-mails at all 115 Acreditation Council for Graduate Medical Education–approved medical doctor neurosurgical training programs in the United States. medical doctor If contact via e-mail yielded no response, another e-mail was sent a week later followed by up to 3 telephone calls and messages. Of 115 programs, we obtained contact information for the chief resident or residents at 108 programs. Three programs asked for the survey link to be distributed through the program director, and 2 programs did not have an administrative chief resident or willing program director. The e-mails were sent out 3 times (temporally spaced 1 week apart) to each chief resident with no reply required (Figure 2).

The results of the survey were analyzed using descriptive statistics with continuous variables summarized through means, SD, and ranges and categorical values as proportions. A total of 25%, 50%, and 75% quartile values were reported when appropriate. Survey response and statistical analysis were performed using Python (v.3.8.7, python.org) and the XM platform (Qualtrics).

## RESULTS

Of 115 programs, 108 programs responded with e-mail contact information to which the survey was distributed. There were a total of 130 respondents with an estimated 9% response rate consisting of 15 PGY-1 (11.54%), 17 PGY-2 (13.08%), 19 PGY-3 (14.62%), 13 PGY-4 (10.00%), 16 PGY-5 (12.31%), 16 PGY-6 (12.31%), and 34 PGY-7 (26.15%).

### Maximum Acceptable Time for Bedside Image Guidance and Preferred Application

**FIGURE 3.**
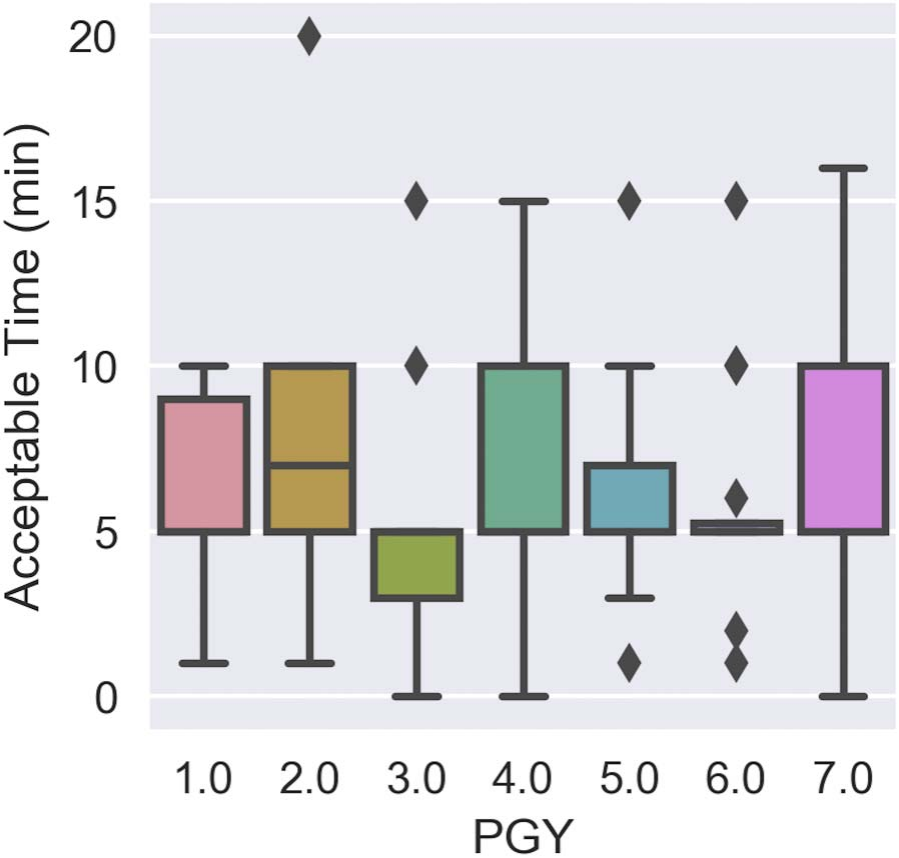
Acceptable time for bedside image guidance in minutes by PGY. The 3 quartile (25%, 50%, 75%) values are shown. The 25% and 50% quartile values are the same (5 min) for all PGY except for PGY-2 and PGY-3. PGY-3 has the same 50% and 75% quartile values at 5 minutes. Plot whiskers extend to points that lie within 1.5 interquartile ranges of the lower and upper quartile, with observations outside of this range displayed independently. PGY, post-graduate year.

The respondents were willing to accept a time addition to their workflow for bedside image guidance ranging from a minimum of 0 min (2 respondents, 2.31%) to a maximum of 20 min (1 respondent, 0.77%) with a mean of 6.39 min (SD = 3.73 min) (Table 1, Figure 3). When analyzing image guidance case preferences, 17 (13.08%) respondents opted to use image guidance for Case 1 with intraventricular hemorrhage and 113 (86.92%) respondents opted to use image guidance for Case 3 with narrow slit-like ventricles. Case 3 was the predominant choice among residents across all years of training.

**TABLE 1.** Average Acceptable Time in Minutes for Bedside Image Guidance by PGY

PGY	No.	Mean	SD	Min	25%	50%	75%	Max
1	15	6.00	3.02	1	5	5	9	10
2	17	7.76	4.13	1	5	7	10	20
3	19	4.74	3.23	0	3	5	5	15
4	13	6.31	3.97	0	5	5	10	15
5	16	6.25	3.38	1	5	5	7	15
6	16	5.88	3.30	1	5	5	5.25	15
7	34	7.15	4.18	0	5	5	10	16

PGY, post-graduate year. The 3 quartile values (25%, 50%, 75%) are shown.

### Reported Number of EVD Misplacements Throughout the Resident Career

**FIGURE 4.**
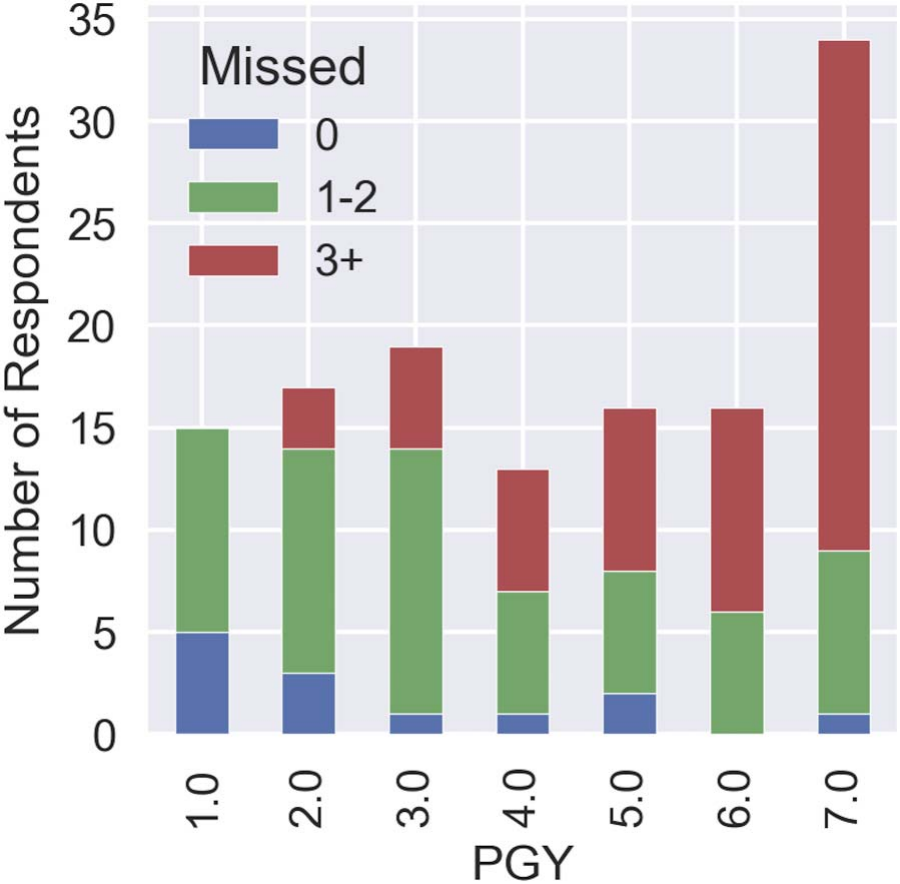
Number of misplaced EVDs by PGY. Approximately 74% of PGY-7 respondents misplaced more than 3 EVDs in their career. EVD, external ventricular drain; PGY, post-graduate year.

Overall, 60 residents (46.15%) misplaced EVDs 1 to 2 times, 57 residents (43.85%) misplaced EVDs 3+ times, and 13 residents (10.00%) did not misplace any EVD throughout their career according to their self-report (Table 2, Figure 4). Residents in PGY-1 had the highest proportion of no misplacements (38.46%), whereas residents in PGY-7 had the highest proportion of 3 or more EVD placements (43.86%).

**TABLE 2.** Number of Misplaced EVDs by PGY

PGY	0	1-2	3+
1	5 (38.46)	10 (16.67)	0
2	3 (23.08)	11 (18.33)	3 (5.26)
3	1 (7.69)	13 (21.67)	5 (8.77)
4	1 (7.69)	6 (10.00)	6 (10.53)
5	2 (15.38)	6 (10.00)	8 (14.04)
6	0	6 (10.00)	10 (17.54)
7	1 (7.69)	8 (13.33)	26 (43.86)
Total	13	60	58

EVD, external ventricular drain; PGY, post-graduate year.

Proportions (%) within each column are given in parentheses.

### Resident Attitudes on the Acceptable Number of EVD Passes

Fifteen (11.54%) respondents believed that only a single pass is acceptable for EVD placements. Seventy-eight (60.00%) respondents believed that up to 2 additional passes are acceptable for EVD placements. Thirty-seven (28.46%) respondents believed that 3 or more additional passes are acceptable for EVD placements (Table 3). Across all resident years, the average number of acceptable passes was at least 2.

**TABLE 3.** Acceptable Number of EVD Passes by PGY

PGY	0	1-2	3+	Mean	SD
1	3 (20.00)	9 (11.54)	3 (8.11)	2.00	0.65
2	1 (6.67)	15 (19.23)	1 (2.70)	2.00	0.35
3	2 (13.33)	12 (15.38)	5 (13.51)	2.16	0.60
4	0	7 (8.97)	6 (16.22)	2.46	0.52
5	3 (20.00)	9 (11.54)	4 (10.81)	2.06	0.68
6	1 (6.67)	7 (8.97)	8 (21.62)	2.44	0.63
7	5 (33.33)	19 (24.36)	10 (27.03)	2.15	0.66
Total	15	78	37	2.17	0.61

EVD, external ventricular drain; PGY, post-graduate year.

A “0” acceptable number of EVD passes refers to no missed passes and that only a single pass is acceptable. Proportions (%) within each column are given in parentheses.

## DISCUSSION

Our survey suggests that residents have limited time tolerance when considering whether to use technological adjuncts for EVD placement at the bedside. A mere 6 minutes of acceptable additional time is drastically faster than approximately 30 to 40 minutes^15,17,19,26^ reported for current technologies. This discrepancy highlights the time pressure placed on residents performing these procedures and may also explain why image guidance is not consistently used for EVD placement despite known benefits in reducing suboptimal positioning.^24,27-29^ When investigating acceptable time by resident year, those in the PGY-3 group tolerated the least amount of additional time on average (4.74 min), perhaps because of a growing number of new teaching and management responsibilities. Besides conventional optical and electromagnetic neuronavigation systems, we discuss the technical nuances, limitations, and benefits of newer commercially available and experimental EVD technological adjuncts and their potential for immediate use at the bedside. For an expanded discussion of neuronavigation, justification of technology, pathways to implementation, and comparison with previous surveys, see **Supplemental Digital Content 2** (http://links.lww.com/NS9/A31).

### Current Image Guidance Technologies

Optical and electromagnetic neuronavigation systems offer high precision of less than 3 to 4 mm^19,30,31^ although these are constrained by large equipment requirements, long setup time, and necessity of head immobilization for infrared tracking systems. Given the limitations of conventional neuronavigation systems, new devices, registration algorithms, and clinical workflows are being explored. Optical topographic imaging navigation systems rapidly acquire hundreds of thousands of surface points using an overhead machine vision camera system with registration times substantially shorter than conventional systems.^32,33^ The FLASH navigation system (SeaSpine) is a commercially available system that involves an overhead camera gantry integrated with light-emitting diodes, a proprietary machine vision camera system and projector, lasers to assist with aiming at the anatomic region of interest for registration, and an infrared optical tracking system.^34^ Registration involves digitization of the intraoperative surface of interest. Initial registration and reregistration occur within 30 and 10 seconds, respectively, greatly surpassing the 15 to 30 minutes of registration time required in other systems. Besides the benefit of faster registration times, integration of the camera with an overhead light source allows the surgeon to control the camera while maintaining sterility and reduces the likelihood of line-of-sight obstructions. Although registration time has significantly improved, this system still requires head immobilization, which can be a large barrier for usage at the bedside.

Near instantaneous registration algorithms have also been reported more recently in the literature. In 2021, Robertson et al^35^ introduced novel surface-based algorithms using global point clouds demonstrating rapid initial registration times of 3 to 5 seconds. The preregistration workflow involves the conversion of a CT scan to a 3-dimensional (3D) mesh and the use of a 3D camera to create a face depth map. The 2-point clouds are then aligned based on area similarity using an artificial intelligence model. Although the reported registration time is significantly faster than older technologies, high speeds are enabled by substantial computing power. The reported registration time also does not account for the time taken to generate preregistration point clouds from the CT scan and 3D video stream of the patient's face. While this could be performed before the procedure in a planned situation, these steps could be barriers for implementation during an urgent bedside procedure. Additional limitations include high cost, processing, and hardware requirements.

Another limitation of existing neuronavigation systems is that the operator must divert their attention away from the surgical field to view the monitor for the current instrument location. Augmented reality (AR) systems have attempted to address this challenge by integrating the line of sight using wearable technology to display an overlay of internal anatomy on the actual surface in view. Li et al in 2018^19^ reported the use of a HoloLens (Microsoft) to guide EVD placement in 15 individuals. The clinical workflow involved manual segmentation of the ventricles from preoperative imaging data, trajectory planning, and export and rendering of the models for access by the HoloLens headset. This took an average of 40 minutes before the surgical procedure although it seemed to improve target deviation compared with the freehand group (4.3 vs 11.3 mm). Manual registration and learning curve for surgical planning in the software environment were significant limitations. Schneider et al in 2021^36^ also reported the use of AR for EVD guidance on a head model although the ventricles were completely missed in 32% of placements and the average deviation between reference trajectory was 7.1 ± 4.1 mm. They note that a limited number of participants were familiar with the AR technology although several participants demonstrated improvement in hit rate, suggesting that an initial learning curve contributed to the high miss rates on the first attempts. Other limitations include the ergonomics of the wearable device, and several participants noted head and nose discomfort when having to wear the device with the operating room mask.

Finally, ultrasound, though not a new concept, has been proposed as a solution for immediate image-guided feedback without the need for registration. Ultrasound guidance has been used extensively at the bedside for non-neurosurgical indications, and thus, the system has already been through multiple design iterations. Early usage of ultrasound involved placing the probe against an open fontanelle.^37,38^ The development of compact transducer tips allowed for its placement against the dura through an expanded burr hole, enabling continuous monitoring.^39-41^ Although the equipment is portable and setup time is minimal, the operator must manipulate both the catheter and ultrasound during the procedure. However, a workaround for this is possible by mounting a guide channel onto the ultrasound transducer as demonstrated by Manfield et al.^21^ While in the United Kingdom, ultrasound guidance is becoming the standard of care for EVD insertion, this has not been popularized in the United States, perhaps because of specialized training required to manipulate the probe and interpret the images, perceptions of a steep learning curve, lack of appropriate probe sizes, and relatively restricted field of view.

### Comparison With Previous Surveys

The fact that our survey agrees with findings from surveys administered more than a decade ago^2,9^ suggests that there may still be technological or logistical barriers that hinder use or that attending preferences toward the freehand approach largely influence EVD image guidance usage among current residents. Limitations in the existing systems may lead to less-than-ideal scenarios being propagated as “acceptable.” In their first year of training, 80% of PGY-1 respondents believed that more than a single pass was acceptable. This comes as no surprise. In recognition of the obvious limitations of the freehand technique, standard practice in neurosurgery has normalized up to 2 passes as satisfactory despite each pass potentially increasing the risk of infection and complications.^6-8^ However, if a medical error is defined as any act resulting in deviation from the perfect course for the patient,^21^ the 2nd pass is a medical error, as is EVD misplacement. In parallel to advancements in technology, stronger efforts should be made to encourage the use of these technologies to provide the best course for the patient.

### Future Directions

Although the benefits of neuronavigation for EVD placement are known, there are significant challenges to its practical application and routine resident usage. Low tolerance for long neuronavigation setup times calls for technologies that are simple for the user and specific to the needs of EVD placement. Current navigation systems may offer nearly perfect catheter placement but can be clunky with limited portability and extensive workflows that add a significant amount of time to the procedure. Of existing systems, electromagnetic neuronavigation and ultrasound-based systems seem to be most amenable to bedside usage. However, there is still a clear need for a user-friendly, portable, rapidly registering navigation method to guide the placement of EVDs.

### Limitations

One of our limitations was our relatively low response rate. Assuming that our survey did make it to all residents in the 108 programs and assuming that there are approximately 14 residents per program (NRMP, 2020), it was estimated that our survey could have reached up to 1512 residents giving us a response rate of approximately 9%. Part of this low response rate will always be due in large part to the voluntary nature of this type of survey. Despite this, our methodology was able to efficiently capture the highest number of neurosurgical resident respondents in an electronic qualitative survey of EVD practices.^2,30^ Future survey methods could include distribution by program directors or confirming with chief residents that e-mails were sent out to give a more accurate denominator.

Other limitations of this survey include the self-reported nature of responses. Although this is an anonymous survey, residents might have under-reported the number of misplacements because of recall bias. The term misplacement was left undefined in the survey although the meaning of misplacement may vary depending on resident perception. Hypothetical questions could be beneficial in the development of new technologies but may be less helpful in understanding current practices. The scenario described in the question stem could introduce bias toward shorter acceptable time additions because of the expectation that such an ideal system should be capable of performing quickly. The questions in the survey also assumed that time and situation would be the most important factors in determining image guidance usage although other factors such as user interface, cost, accessibility, and attending preferences could contribute.

## CONCLUSION

An ideal EVD neuronavigation system would add not more than 6 minutes on average to the user's workflow. Residents of all training levels seem to prefer neuronavigation for EVD placement in complex cases of slit ventricles, rather than intraventricular hemorrhage or hydrocephalus. Future EVD neuronavigation technologies should focus on efficiency of user experience and techniques to achieve rapid registration.

## SUPPLEMENTAL DIGITAL CONTENT

**Supplemental Digital Content 1. Methods, Questionnaire.** The Supplemental Digital Content expands on the Methods provided. **Supplemental Digital Content 2. Discussion, Expanded.** This Supplemental Content provides a background overview of neuronavigation, discusses the justification of this technology, expands on comparisons of our data with previous surveys, and details pathways to implementation.

## COMMENTS

In the words of W. Edwards Deming, “Every system is perfectly designed to produce the results it gets.” External ventricular drain (EVD) placements are among the most common neurosurgical procedures performed. The freehand technique has predominated for over a century, and while it enables efficiency, this method is susceptible to inaccurate catheter placement, multiple attempts, injury to brain parenchyma, tract hemorrhage, catheter dysfunction, and/or infection.^1a,2a^ In cases where the patient's anatomy heightens the complexity and risk of ventriculostomy, frameless neuronavigation has the potential to decrease patient harm.

The authors' cross-sectional survey of neurosurgical residents' perspectives on EVD placement and associated technology is an important one. Enabling technologies (using computer vision–based surface mapping, electromagnetism, etc.) are being rapidly developed, and it is reasonable to gauge residents' perceived need for these tools and interest in adoption, particularly as the primary end users. The 130 resident responses underscored an openness to use image guidance technology, particularly for narrow slit-like ventricles, as well as a preference for efficiency, with the technology adding less than 6.4 minutes to the workflow.

As a field, we should carefully consider the value equation for these innovations, which incorporates the benefits to the patient (outcomes and experience) in the numerator and costs (time, financial, direct, indirect) in the denominator.^3a^

Regarding patient benefit, not only would frameless neuronavigation facilitate the safe placement of EVD catheters, but also enhanced procedural precision and accuracy could increase opportunities for intraventricular and intracranial interventions. For example, would navigation have affected the results of the CLEAR III trial of tPA for intraventricular thrombolysis, where 21% patients incurred a tract hemorrhage, and 29% of catheters terminated in an undesired location?^4a^ Furthermore, a navigation system's independence from cranial fixation expands its usability beyond conventional operating rooms, whether that is bedside or in a procedure room at an Ambulatory Surgical Center. This adaptability can reduce expenses associated with operating rooms and could include Ommaya reservoir placement, brain biopsy, focused ultrasound for intracranial drug delivery, and more. Every system is perfectly designed to produce the results it gets, and we have duty to evaluate and responsibly integrate enabling technology into our systems.

**Faith Robertson**

*Boston, Massachusetts, USA*
